# The genome sequence of the Willow Beauty, *Peribatodes rhomboidaria* (Denis & Schiffermüller, 1775)

**DOI:** 10.12688/wellcomeopenres.19479.1

**Published:** 2023-06-02

**Authors:** Douglas Boyes, Marianne Eagles, Peter W.H. Holland, Darwin Tree of Life Barcoding collective, Wellcome Sanger Institute Tree of Life programme, Wellcome Sanger Institute Scientific Operations: DNA Pipelines collective, Tree of Life Core Informatics collective, Darwin Tree of Life Consortium

**Affiliations:** 1 UK Centre for Ecology & Hydrology, Wallingford, England, UK; 2 Independent researcher, Crawley Down, England, UK; 3 University of Oxford, Oxford, England, UK

**Keywords:** Peribatodes rhomboidaria, the Willow Beauty, genome sequence, chromosomal, Lepidoptera

## Abstract

We present a genome assembly from an individual male *Peribatodes rhomboidaria* (the Willow Beauty; Arthropoda; Insecta; Lepidoptera; Geometridae). The genome sequence is 499.7 megabases in span. Most of the assembly is scaffolded into 31 chromosomal pseudomolecules including the Z sex chromosome. The mitochondrial genome has also been assembled and is 15.7 kilobases in length. Gene annotation of this assembly on Ensembl identified 18,486 protein coding genes.

## Species taxonomy

Eukaryota; Metazoa; Ecdysozoa; Arthropoda; Hexapoda; Insecta; Pterygota; Neoptera; Endopterygota; Lepidoptera; Glossata; Ditrysia; Geometroidea; Geometridae; Ennominae; *Peribatodes*; *Peribatodes rhomboidaria* (Denis & Schiffermüller, 1775) (NCBI:txid190356).

## Background

The Willow Beauty ( *Peribatodes rhomboidaria*) is a moth in the family Geometridae, a group in which many species have wings patterned with wavy lines, dots, and speckling, providing crypsis when settled with wings flat against the bark of a tree. The Willow Beauty has a pale brown ground colour, dark speckles, and a pair of black transverse lines converging sharply together at the trailing edge of the forewing. The species exhibits variation in ground colour, including a uniformly black melanic form thought to be due to a dominant allele and a grey variant ( Ford, 1967; South, 1961; Waring *et al.*, 2017). The wingspan is typically 35 to 45 mm in mainland Europe or 40 to 48 mm in the UK ( Skinner & Wilson, 2009), with smaller specimens often seen late in the season.

The larvae are polyphagous, feeding on the leaves of many broadleaved plants including hawthorn, privet, ivy and conifers ( Henwood *et al.*, 2020). The common name Willow Beauty, therefore, is a misnomer as the species does not favour willow and is not particularly beautiful. In Italy, France and Romania, *P. rhomboidaria* has been reported as an intermittent pest of vineyards; major damage can be caused by larvae which have overwintered feeding on the new buds of grapevine in spring ( Busato *et al.*, 2000; de Meirleire, 1979; Filip, 1998; Julei, 1984).

This moth is widely distributed throughout Europe, including inland and coastal habitats in western Europe, and is primarily found in coastal locations in Scandinavia and around the Black Sea and Caspian Sea ( GBIF Secretariat, 2022). In the UK, the Willow Beauty is common in gardens, woodlands, and scrub habitats in England and Wales, but less frequently recorded in Scotland ( NBN Atlas Partnership, 2022; Randle *et al.*, 2019).

The flight season and voltinism has been observed to be changing, with one generation per year and adult moths on the wing from June to August in the north of the UK and in Ireland, and a recent increase to two overlapping flight seasons from May to October in southern England ( Waring *et al.*, 2017).

Molecular cytogenetic analyses have revealed a particularly large and heterochromatic W chromosome in females ( Hejníčková *et al.*, 2021), with low-depth genome sequencing and chromosome hybridization showing that the W chromosome is rich in female-enriched DNA repeats ( Hejníčková *et al.*, 2022). The assembled genome sequence for *P. rhomboidaria* will facilitate the study of the genetic basis of cryptic colouration, polymorphism, and polyphagy, and contribute to the growing dataset of genomic resources for understanding lepidopteran biology.

## Genome sequence report

**Figure 1.**
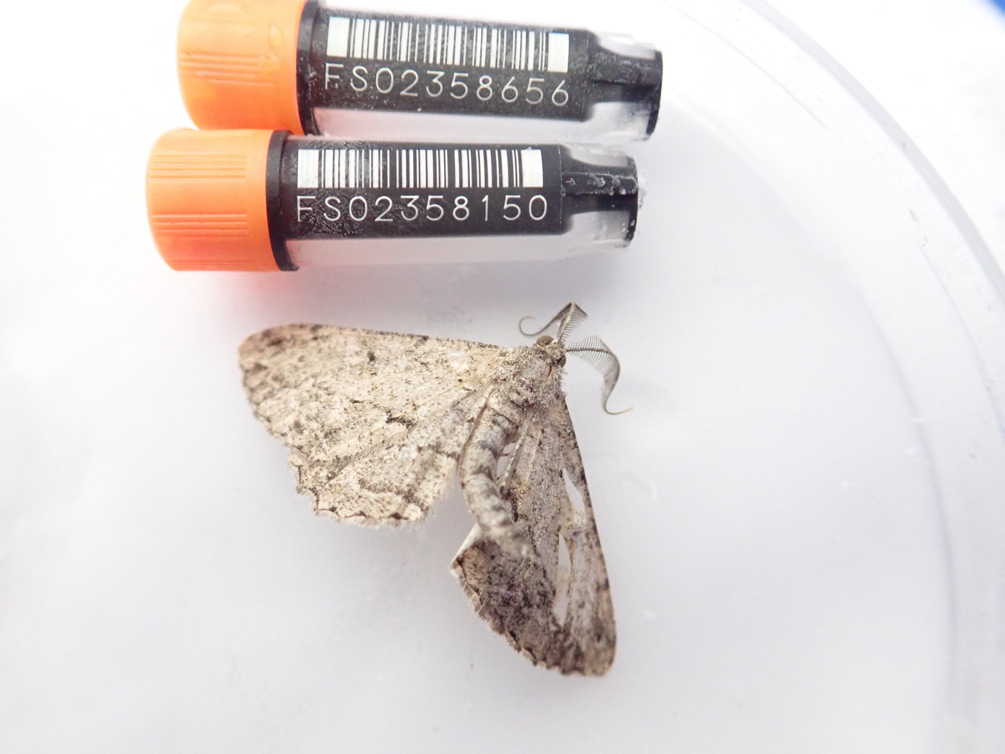
Image of the *Peribatodes rhomboidaria* (ilPerRhom1) specimen used for genome sequencing.

The genome was sequenced from one male *Peribatodes rhomboidaria* ( Figure 1) collected from Wytham Woods, Oxfordshire, UK (51.77, –1.34). A total of 44-fold coverage in Pacific Biosciences single-molecule HiFi long reads and 74-fold coverage in 10X Genomics read clouds were generated. Primary assembly contigs were scaffolded with chromosome conformation Hi-C data. Manual assembly curation corrected 17 missing or mis-joins and removed one haplotypic duplication, reducing the assembly length by 0.17% and the scaffold number by 16.28%.

**Figure 2.**
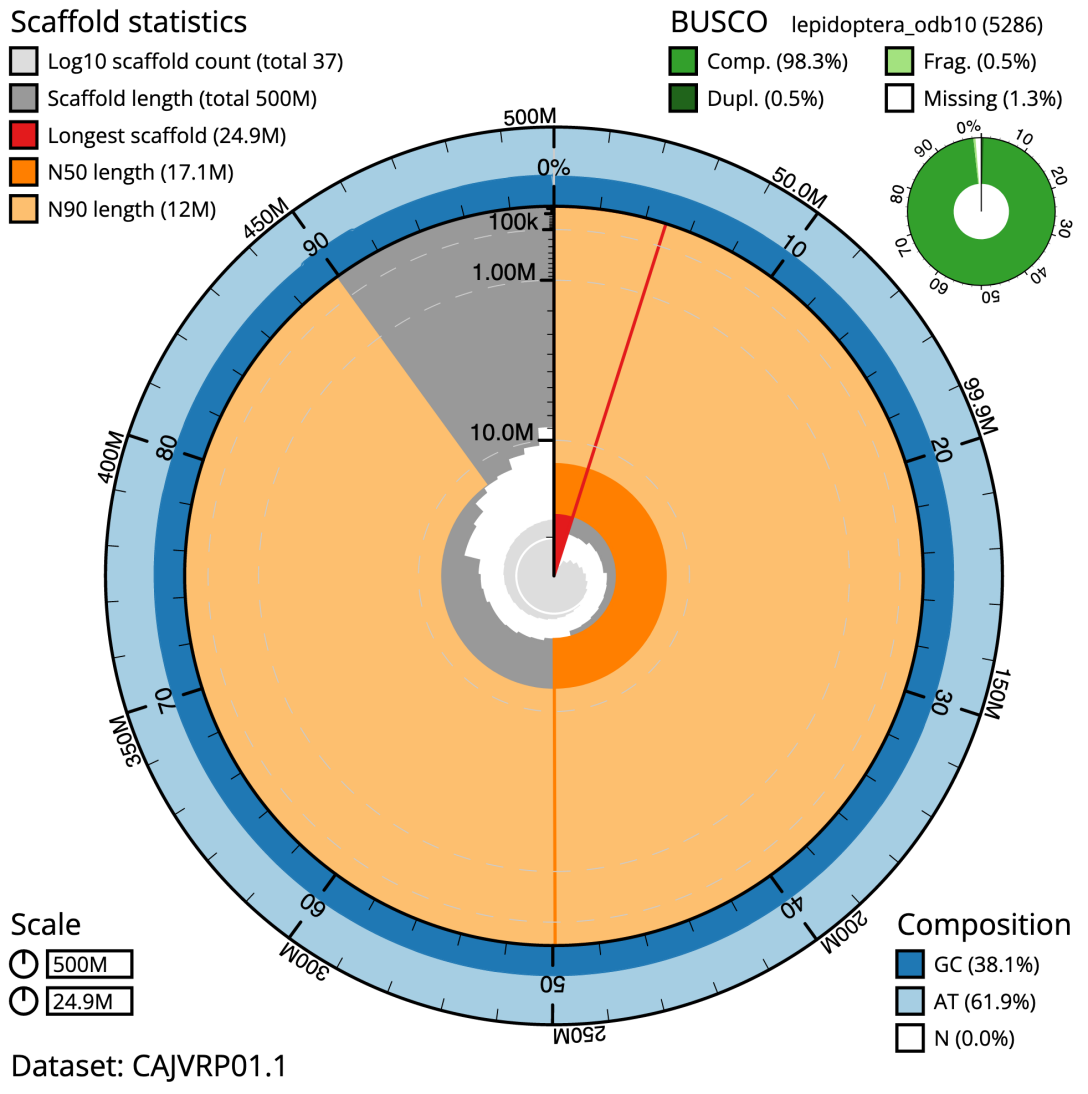
Genome assembly of *Peribatodes rhomboidaria*, ilPerRhom1.1: metrics. The BlobToolKit Snailplot shows N50 metrics and BUSCO gene completeness. The main plot is divided into 1,000 size-ordered bins around the circumference with each bin representing 0.1% of the 499,728,750 bp assembly. The distribution of scaffold lengths is shown in dark grey with the plot radius scaled to the longest scaffold present in the assembly (24,949,269 bp, shown in red). Orange and pale-orange arcs show the N50 and N90 scaffold lengths (17,104,115 and 12,037,098 bp), respectively. The pale grey spiral shows the cumulative scaffold count on a log scale with white scale lines showing successive orders of magnitude. The blue and pale-blue area around the outside of the plot shows the distribution of GC, AT and N percentages in the same bins as the inner plot. A summary of complete, fragmented, duplicated and missing BUSCO genes in the lepidoptera_odb10 set is shown in the top right. An interactive version of this figure is available at https://blobtoolkit.genomehubs.org/view/ilPerRhom1.1/dataset/CAJVRP01.1/snail.

The final assembly has a total length of 499.7 Mb in 36 sequence scaffolds with a scaffold N50 of 17.1 Mb ( Table 1). Most (99.96%) of the assembly sequence was assigned to 31 chromosomal-level scaffolds, representing 30 autosomes and the Z sex chromosome. Chromosome-scale scaffolds confirmed by the Hi-C data are named in order of size ( Figure 2– Figure 5; Table 2). While not fully phased, the assembly deposited is of one haplotype. Contigs corresponding to the second haplotype have also been deposited.

**Table 1.** Genome data for *Peribatodes rhomboidaria*, ilPerRhom1.1.

Project accession data		
Assembly identifier	ilPerRhom1.1	
Species	*Peribatodes rhomboidaria*	
Specimen	ilPerRhom1	
NCBI taxonomy ID	190356	
BioProject	PRJEB45204	
BioSample ID	SAMEA7701524	
Isolate information	ilPerRhom1, male; abdomen (DNA sequencing), head and thorax (Hi-C scaffolding)	
Assembly metrics *		*Benchmark*
Consensus quality (QV)	59.8	*≥ 50*
*k*-mer completeness	100%	*≥ 95%*
BUSCO **	C:98.3%[S:97.8%,D:0.5%], F:0.5%,M:1.3%,n:5,286	*C ≥ 95%*
Percentage of assembly mapped to chromosomes	99.96%	*≥ 95%*
Sex chromosomes	Z chromosome	*localised homologous pairs*
Organelles	Mitochondrial genome assembled	*complete single alleles*
Raw data accessions		
PacificBiosciences SEQUEL II	ERR6412045, ERR6454733	
10X Genomics Illumina	ERR6055002–ERR6055005	
Hi-C Illumina	ERR6055001	
Genome assembly		
Assembly accession	GCA_911728515.1	
*Accession of alternate haplotype*	GCA_911728505.1	
Span (Mb)	499.7	
Number of contigs	59	
Contig N50 length (Mb)	15.1	
Number of scaffolds	36	
Scaffold N50 length (Mb)	17.1	
Longest scaffold (Mb)	25	
Genome annotation		
Number of protein-coding genes	18,486	
Number of gene transcripts	18,692	

* Assembly metric benchmarks are adapted from column VGP-2020 of “Table 1: Proposed standards and metrics for defining genome assembly quality” from ( Rhie *et al.*, 2021). ** BUSCO scores based on the lepidoptera_odb10 BUSCO set using $BUSCO_VERSION. C = complete [S = single copy, D = duplicated], F = fragmented, M = missing, n = number of orthologues in comparison. A full set of BUSCO scores is available at https://blobtoolkit.genomehubs.org/view/ilPerRhom1.1/dataset/CAJVRP01.1/busco.

**Figure 3.**
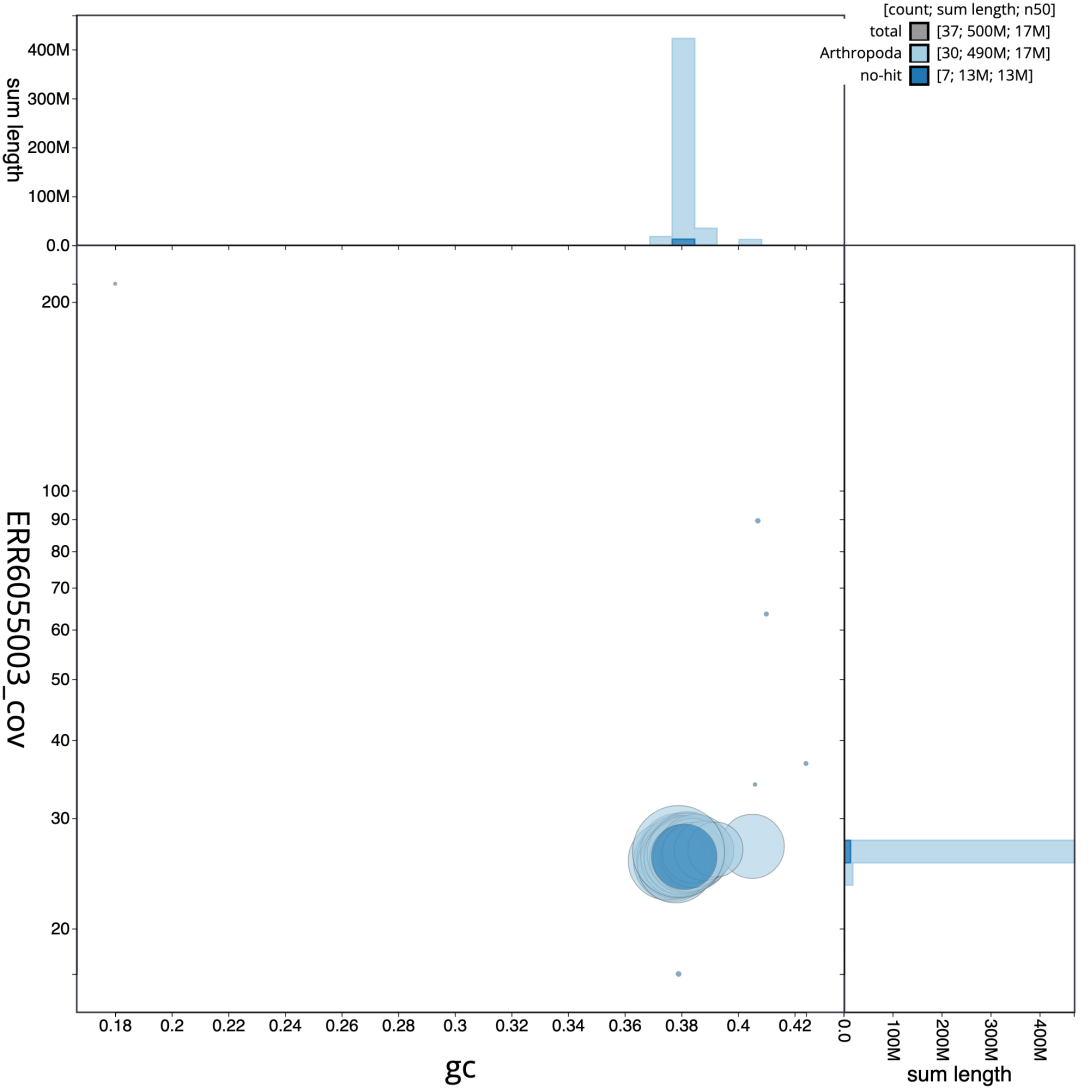
Genome assembly of *Peribatodes rhomboidaria*, ilPerRhom1.1: GC coverage. BlobToolKit GC-coverage plot. Scaffolds are coloured by phylum. Circles are sized in proportion to scaffold length. Histograms show the distribution of scaffold length sum along each axis. An interactive version of this figure is available at https://blobtoolkit.genomehubs.org/view/ilPerRhom1.1/dataset/CAJVRP01.1/blob.

**Figure 4.**
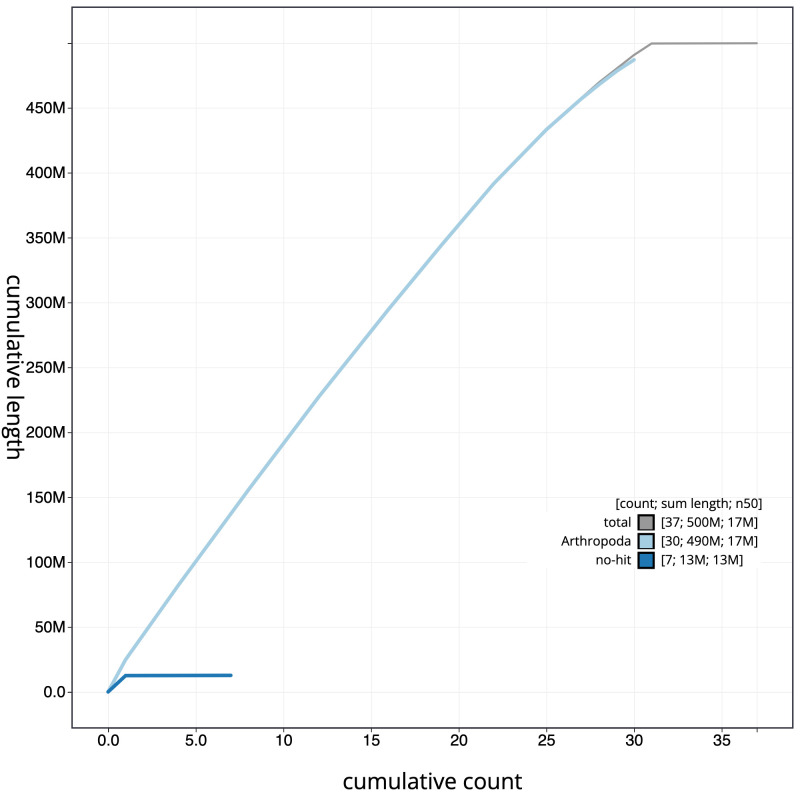
Genome assembly of *Peribatodes rhomboidaria*, ilPerRhom1.1: cumulative sequence. BlobToolKit cumulative sequence plot. The grey line shows cumulative length for all scaffolds. Coloured lines show cumulative lengths of scaffolds assigned to each phylum using the buscogenes taxrule. An interactive version of this figure is available at https://blobtoolkit.genomehubs.org/view/ilPerRhom1.1/dataset/CAJVRP01.1/cumulative.

**Figure 5.**
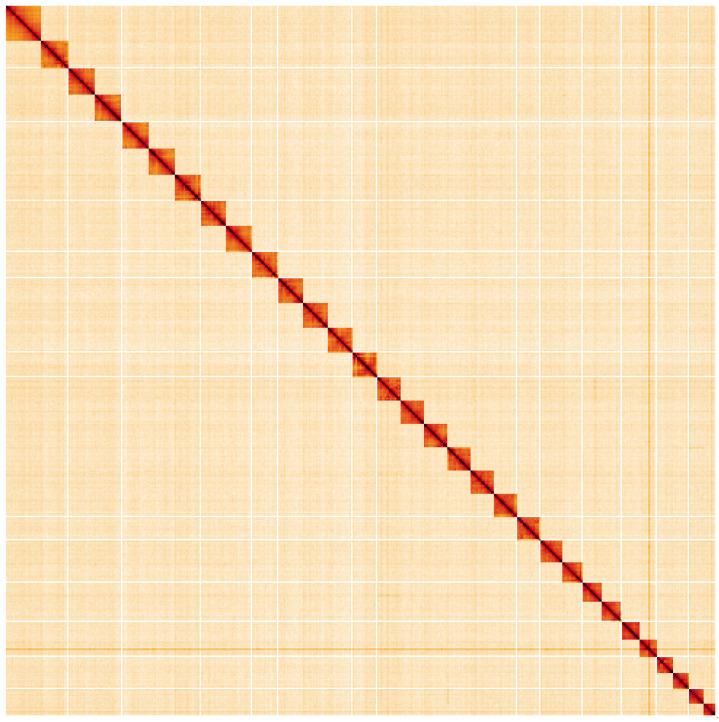
Genome assembly of *Peribatodes rhomboidaria*, ilPerRhom1.1: Hi-C contact map. Hi-C contact map of the ilPerRhom1.1 assembly, visualised using HiGlass. Chromosomes are shown in order of size from left to right and top to bottom. An interactive version of this figure may be viewed at https://genome-note-higlass.tol.sanger.ac.uk/l/?d=Sa1ZSyVfQ96U6daRyLhLOA,

**Table 2.** Chromosomal pseudomolecules in the genome assembly of *Peribatodes rhomboidaria*, ilPerRhom1.

INSDC accession	Chromosome	Size (Mb)	GC%
OU452167.1	1	19.23	38.2
OU452168.1	2	19.14	37.9
OU452169.1	3	18.73	38.2
OU452170.1	4	18.64	37.9
OU452171.1	5	18.59	38
OU452172.1	6	18.18	38.2
OU452173.1	7	18.12	37.7
OU452174.1	8	18.03	38
OU452175.1	9	17.91	37.7
OU452176.1	10	17.79	37.5
OU452177.1	11	17.71	37.8
OU452178.1	12	17.12	37.8
OU452179.1	13	17.1	37.9
OU452180.1	14	16.82	38.1
OU452181.1	15	16.72	37.9
OU452182.1	16	16.47	38.1
OU452183.1	17	16.36	38
OU452184.1	18	16.35	38
OU452185.1	19	16.07	38.1
OU452186.1	20	15.91	38.4
OU452187.1	21	15.69	38.5
OU452188.1	22	13.98	38.1
OU452189.1	23	13.97	38.4
OU452190.1	24	13.61	38.2
OU452191.1	25	12.53	38.1
OU452192.1	26	12.04	40.5
OU452193.1	27	11.81	38.4
OU452194.1	28	10.84	38.4
OU452195.1	29	10.31	38.8
OU452196.1	30	8.82	39.2
OU452166.1	Z	24.95	37.9
OU452197.1	MT	0.02	18.1
-	unplaced	0.19	40.4

The mitochondrial genome was also assembled and can be found as a contig within the multifasta file of the genome submission.

The estimated Quality Value (QV) of the final assembly is 59.8 with *k*-mer completeness of 100%, and the assembly has a BUSCO v5.3.2 completeness of 98.3% (single = 97.8%, duplicated = 0.5%), using the lepidoptera_odb10 reference set ( *n* = 5,286).

Metadata for specimens, spectral estimates, sequencing runs, contaminants and pre-curation assembly statistics can be found at https://links.tol.sanger.ac.uk/species/190356.

## Genome annotation report

The *P. rhomboidaria* GCA_911728515.1 genome assembly was annotated using the Ensembl rapid annotation pipeline ( Table 1; https://rapid.ensembl.org/Peribatodes_rhomboidaria_GCA_911728515.1/). The resulting annotation includes 18,692 transcribed mRNAs from 18,486 protein-coding genes.

## Methods

### Sample acquisition and nucleic acid extraction

A male *Peribatodes rhomboidaria* (specimen number Ox000663, individual ilPerRhom1) was collected from Wytham Woods, Oxfordshire (biological vice-county: Berkshire), UK (latitude 51.77, longitude –1.34) on 20 July 2020. The specimen was collected and identified by Douglas Boyes (University of Oxford). The specimen was taken from woodland habitat using a light trap. The specimen was snap-frozen on dry ice.

DNA was extracted at the Tree of Life laboratory, Wellcome Sanger Institute (WSI). The ilPerRhom1 sample was weighed and dissected on dry ice with tissue set aside for Hi-C sequencing. Abdomen tissue was disrupted using a Nippi Powermasher fitted with a BioMasher pestle. High molecular weight (HMW) DNA was extracted using the Qiagen MagAttract HMW DNA extraction kit. Low molecular weight DNA was removed from a 20 ng aliquot of extracted DNA using 0.8X AMpure XP purification kit prior to 10X Chromium sequencing; a minimum of 50 ng DNA was submitted for 10X sequencing. HMW DNA was sheared into an average fragment size of 12–20 kb in a Megaruptor 3 system with speed setting 30. Sheared DNA was purified by solid-phase reversible immobilisation using AMPure PB beads with a 1.8X ratio of beads to sample to remove the shorter fragments and concentrate the DNA sample. The concentration of the sheared and purified DNA was assessed using a Nanodrop spectrophotometer and Qubit Fluorometer and Qubit dsDNA High Sensitivity Assay kit. Fragment size distribution was evaluated by running the sample on the FemtoPulse system.

### Sequencing

Pacific Biosciences HiFi circular consensus and 10X Genomics read cloud DNA sequencing libraries were constructed according to the manufacturers’ instructions. DNA sequencing was performed by the Scientific Operations core at the WSI on Pacific Biosciences SEQUEL II (HiFi) and Illumina NovaSeq 6000 (10X) instruments. Hi-C data were also generated from head and thorax tissue of ilPerRhom1 using the Arima2 kit and sequenced on the Illumina NovaSeq 6000 instrument.

### Genome assembly

Assembly was carried out with Hifiasm ( Cheng *et al.*, 2021) and haplotypic duplication was identified and removed with purge_dups ( Guan *et al.*, 2020). One round of polishing was performed by aligning 10X Genomics read data to the assembly with Long Ranger ALIGN, calling variants with freebayes ( Garrison & Marth, 2012). The assembly was then scaffolded with Hi-C data ( Rao *et al.*, 2014) using SALSA2 ( Ghurye *et al.*, 2019). The assembly was checked for contamination as described previously ( Howe *et al.*, 2021). Manual curation was performed using HiGlass ( Kerpedjiev *et al.*, 2018) and Pretext ( Harry, 2022). The mitochondrial genome was assembled using MitoHiFi ( Uliano-Silva *et al.*, 2022), which performed annotation using MitoFinder ( Allio *et al.*, 2020). The genome was analysed and BUSCO scores generated within the BlobToolKit environment ( Challis *et al.*, 2020). Table 3 contains a list of all software tool versions used, where appropriate.

**Table 3.** Software tools: versions and sources.

Software tool	Version	Source
BlobToolKit	3.5.2	https://github.com/blobtoolkit/blobtoolkit
BUSCO	5.3.2	https://gitlab.com/ezlab/busco
FreeBayes	1.3.1-17-gaa2ace8	https://github.com/freebayes/freebayes
Hifiasm	0.15	https://github.com/chhylp123/hifiasm
HiGlass	1.11.6	https://github.com/higlass/higlass
Long Ranger ALIGN	2.2.2	https://support.10xgenomics.com/genome-exome/ software/pipelines/latest/advanced/other-pipelines
Merqury	MerquryFK	https://github.com/thegenemyers/MERQURY.FK
MitoHiFi	2	https://github.com/marcelauliano/MitoHiFi
PretextView	0.2	https://github.com/wtsi-hpag/PretextView
purge_dups	1.2.3	https://github.com/dfguan/purge_dups
SALSA	2.2	https://github.com/salsa-rs/salsa
sanger-tol/genomenote	v1.0	https://github.com/sanger-tol/genomenote
sanger-tol/readmapping	1.1.0	https://github.com/sanger-tol/readmapping/tree/1.1.0

### Genome annotation

The BRAKER2 pipeline ( Brůna *et al.*, 2021) was used in the default protein mode to generate annotation for the *P. rhomboidaria* assembly (GCA_934047225.1) in Ensembl Rapid Release.

### Legal and ethical review process for Darwin Tree of Life Partner submitted materials

The materials that have contributed to this genome note have been supplied by a Darwin Tree of Life Partner.

The submission of materials by a Darwin Tree of Life Partner is subject to the **‘Darwin Tree of Life Project Sampling Code of Practice’**, which can be found in full on the Darwin Tree of Life website here. By agreeing with and signing up to the Sampling Code of Practice, the Darwin Tree of Life Partner agrees they will meet the legal and ethical requirements and standards set out within this document in respect of all samples acquired for, and supplied to, the Darwin Tree of Life Project.

Further, the Wellcome Sanger Institute employs a process whereby due diligence is carried out proportionate to the nature of the materials themselves, and the circumstances under which they have been/are to be collected and provided for use. The purpose of this is to address and mitigate any potential legal and/or ethical implications of receipt and use of the materials as part of the research project, and to ensure that in doing so we align with best practice wherever possible.

The overarching areas of consideration are:

- Ethical review of provenance and sourcing of the material
- Legality of collection, transfer and use (national and international)

Each transfer of samples is further undertaken according to a Research Collaboration Agreement or Material Transfer Agreement entered into by the Darwin Tree of Life Partner, Genome Research Limited (operating as the Wellcome Sanger Institute), and in some circumstances other Darwin Tree of Life collaborators.

## Data Availability

European Nucleotide Archive: *Peribatodes rhomboidaria* (willow beauty). Accession number PRJEB45204; https://identifiers.org/ena.embl/PRJEB45204. ( Wellcome Sanger Institute, 2021) The genome sequence is released openly for reuse. The *Peribatodes rhomboidaria* genome sequencing initiative is part of the Darwin Tree of Life (DToL) project. All raw sequence data and the assembly have been deposited in INSDC databases. Raw data and assembly accession identifiers are reported in Table 1.

## Author information

Members of the University of Oxford and Wytham Woods Genome Acquisition Lab are listed here: https://doi.org/10.5281/zenodo.4789928.

Members of the Darwin Tree of Life Barcoding collective are listed here: https://doi.org/10.5281/zenodo.4893703.

Members of the Wellcome Sanger Institute Tree of Life programme are listed here: https://doi.org/10.5281/zenodo.4783585.

Members of Wellcome Sanger Institute Scientific Operations: DNA Pipelines collective are listed here: https://doi.org/10.5281/zenodo.4790455.

Members of the Tree of Life Core Informatics collective are listed here: https://doi.org/10.5281/zenodo.5013541.

Members of the Darwin Tree of Life Consortium are listed here: https://doi.org/10.5281/zenodo.4783558.
