# Supplementary material for: The genome sequence of the Willow Beauty, Peribatodes rhomboidaria (Denis & Schiffermüller, 1775)
Source: Wellcome Open Res. 2023 Jun 2;8:233. [Version 1] doi: 10.12688/wellcomeopenres.19479.1 (PMC10799229; doi:10.12688/wellcomeopenres.19479.1)
Supplement: Peer review history

# Reviewer report: Reviewer response for version 1

**DOI:** 10.21956/wellcomeopenres.21577.r71176

**Published:** 2024-01-19

**Authors:** Puteri Nur Syahzanani Jahari

**Affiliations:** 1 Department of Biosciences, Faculty of Science, Universiti Teknologi Malaysia, Skudai, Johor, Malaysia

The article is well-written. The background study of the species is also well described. I only have one minor comment that the author may or may not agree with.

 - Background section: "... therefore, is a misnomer as the species does not favour willow and is not particularly beautiful" shall be removed. I think the beauty of a species is subjective. The author may not see it as beautiful but others may love the physique of the species.

Are sufficient details of methods and materials provided to allow replication by others?

Yes

Is the rationale for creating the dataset(s) clearly described?

Yes

Are the datasets clearly presented in a useable and accessible format?

Yes

Are the protocols appropriate and is the work technically sound?

Yes

Reviewer Expertise:

Mitogenomics, DNA barcoding, basic evolutionary

I confirm that I have read this submission and believe that I have an appropriate level of expertise to confirm that it is of an acceptable scientific standard.

# Reviewer report: Reviewer response for version 1

**DOI:** 10.21956/wellcomeopenres.21577.r59457

**Published:** 2023-06-27

**Authors:** Omaththage P. Perera

**Affiliations:** 1 Southern Insect Management Research Unit, USDA, Agricultural Research Service, Stoneville, MS, USA

The genome assembly report for the Willow Beauty is well written and describes in detail the process of sequencing and assembling the genome. The authors use a "well oiled" pipeline to assemble this high quality genome. The couple of minor suggestions that authors may or may not accept are listed below:

- In the sentence ":The common name Willow Beauty, therefore, is a misnomer as the species does not favour willow and is not particularly beautiful" , the part that state "...and is not particularly beautiful" should be removed. Beauty is in the eyes of the beholder, and we do not know the etymology of the name. Therefore, I believe that pointing out that this insect does not favour Willow is sufficient. 
- The sentence "The genome was sequenced from one male *Peribatodes rhomboidaria* (Figure 1) collected from Wytham Woods, Oxfordshire, UK (51.77, –1.34)" in the Genome Sequence Report section and the sentence "A male *Peribatodes rhomboidaria* (specimen number Ox000663, individual ilPerRhom1) was collected from Wytham Woods, Oxfordshire (biological vice-county: Berkshire), UK (latitude 51.77, longitude –1.34) on 20 July 2020" in the methods section duplicates the same information and may be revised.

Are sufficient details of methods and materials provided to allow replication by others?

Yes

Is the rationale for creating the dataset(s) clearly described?

Yes

Are the datasets clearly presented in a useable and accessible format?

Yes

Are the protocols appropriate and is the work technically sound?

Yes

Reviewer Expertise:

Genetics, molecular biology, population genetics

I confirm that I have read this submission and believe that I have an appropriate level of expertise to confirm that it is of an acceptable scientific standard.
